# Nucleation and growth of TiAl_3_ intermetallic phase in diffusion bonded Ti/Al Metal Intermetallic Laminate

**DOI:** 10.1038/s41598-018-35247-0

**Published:** 2018-11-14

**Authors:** N. Thiyaneshwaran, K. Sivaprasad, B. Ravisankar

**Affiliations:** Advanced Materials Processing Laboratory Department of Metallurgical & Materials Engineering, National Institute of Technology Tiruchirappalli, Tamil Nadu, 620015 India

## Abstract

A novel nucleation and growth phenomenon for TiAl_3_ intermetallic phase in Ti/Al diffusion couple is proposed based on diffusion kinetics. The interdiffusion and intrinsic diffusion co-efficients are calculated to make evident of dominant diffusion of Al towards Ti in Ti/Al diffusion couple obtained by solid state diffusion bonding. It was surprising to observe that the diffusion rate of Al was around 20 times higher than Ti with the formation of Kirkendall pores near the Al/TiAl_3_ interface. With such dominant diffusion of Al towards Ti, the nucleation and growth of TiAl_3_ intermetallic phase in Ti/Al couple happens mainly at the Ti/TiAl_3_ interface rather than Al/TiAl_3_ interface which is evident by the presence of very fine nearly nano-sized TiAl_3_ nuclei/grains near the Ti/TiAl_3_ interface. Even though the intermetallic phase is expected to nucleate at Al/TiAl_3_ interface, the relatively larger TiAl_3_ grains near that interface depicts grain growth with minimal nucleation. The theoretical calculations on diffusion parameters are in accordance with experimental observations of TiAl_3_ intermetallic growth phenomenon in Ti/Al system.

## Introduction

Ti/TiAl_3_/Al metal intermetallic laminates (MILs) are considered to be one of the promising materials for defense and aerospace applications due to its combined properties such as lower density, high specific strength and relatively good oxidation and corrosion resistance^1–4^. In these MILs, TiAl_3_ is the only intermetallic phase that formed between Ti and Al at relatively lower temperatures (below Al melting point) as other intermetallic phases such as Ti_3_Al, TiAl and TiAl_2_ are expected to form either at higher temperatures or after the consumption of Al^5^. The nucleation and growth of TiAl_3_ intermetallic phase (below Al melting temperature) in the Ti/Al system mostly depends on the interdiffusion and relative rates of diffusion of Ti and Al atoms as well on thermodynamic factors. Almost, the studies conducted on Ti/Al system depicts, Al to be the dominant diffusing species at temperatures below Al melting point while Ti was considered to diffuse faster at higher temperatures especially above Al melting temperature^6–9^. This led to the conclusion that TiAl_3_ nucleates due to the high diffusion of Al towards Ti and the nucleation of TiAl_3_ phase should be concentrated mostly at Ti/TiAl_3_ interface. In contrast, Xu *et al*.^10^ observed the diffusion of both Al and Ti through TiAl_3_ layer in the Ti/Al system regardless of the bonding temperature (below or above Al melting point) employed, emphasizing the growth of TiAl_3_ on both Ti/TiAl_3_ and Al/TiAl_3_ interfaces. It is also assumed that the solid solution formation of Al(Ti) preceding the TiAl_3_ nucleation on Al/TiAl_3_ interface helps in the faster nucleation of TiAl_3_ nuclei at Al/TiAl_3_ side due to less solubility of Ti in Al^10,11^. On the other hand, Mirjalili *et al*.^9^ observed dominant diffusion of Al towards Ti layer and formation of fine equiaxed TiAl_3_ grains near Ti/TiAl_3_ interface, claiming dominant nucleation of TiAl_3_ nuclei at the Ti/TiAl_3_ interface. While at the Al/TiAl_3_ interface, coarse grains were observed indicating the growth of initial TiAl_3_ grains than nucleation. It is now obvious that there exist contradictory viewpoints or observations regarding the nucleation and growth of TiAl_3_ phase and there are no supporting interdiffusion parameter calculations to validate dominant diffusion of Al atoms in Ti/Al system. As the nucleation and growth of TiAl_3_ is also dependent on the diffusion kinetics, the diffusion parameter calculation helps in understanding the nucleation and growth phenomenon in the system.

Hence, the present work is mainly focused on calculating interdiffusion co-efficient of Ti and Al and thereby to clarify the contradictions in nucleation and growth phenomenon of TiAl_3_ intermetallic phase in multi-laminated Ti/Al diffusion couple.

## Methods

Ti/TiAl_3_/Al MILs were prepared using commercially pure Ti and Al sheets of 0.5 mm thickness through solid state diffusion bonding technique. The bonding was carried out at temperatures 550 and 575 °C for bonding times of 2, 4 and 6 h in vacuum (−760 mm of Hg). The sheets were rinsed with 10% HF solution and ultrasonically cleaned prior to bonding to remove surface oxides and impurities. The cleaned sheets were stacked alternatively in the bonding setup and a uniaxial pressure of 4 MPa was applied throughout the bonding time to prepare MILs with a total thickness of 5 mm. To analyse the growth of the intermetallic phases on Ti/TiAl_3_ and Al/TiAl_3_ interfaces, the 6 h bonded MILs already containing a specific intermetallic phase along the Ti/Al interface, were again annealed for 12, 24, 36 and 48 h duration at their respective bonding temperatures without external pressure. A brief schematic of the sample preparation process can be found elsewhere (See Supplementary Fig. S1). The microstructural characterization and phase analysis were performed on the cross section of the MILs using Hitachi S3000H scanning electron microscope (SEM) attached with Thermo USA energy dispersive spectroscopy (EDS). X-Ray diffraction (XRD) study was performed on the annealed sample to analyse the phases formed in the intermetallic region. Totally three samples in each condition are used to analyse the results.

The diffusion parameters such as interdiffusion co-efficient and tracer diffusion co-efficient were calculated using Wagner’s method^12^ in the MILs annealed for different duration. The nucleation and growth phenomenon of TiAl_3_ phase at Ti/TiAl_3_ and Al/TiAl_3_ interfaces are analysed based on the grain size distribution of TiAl_3_, obtained through orientation imaging microscopy (OIM) micrographs taken using electron backscattered diffraction (EBSD) technique.

## Results

### Microstructure Evolution

**Figure 1:**
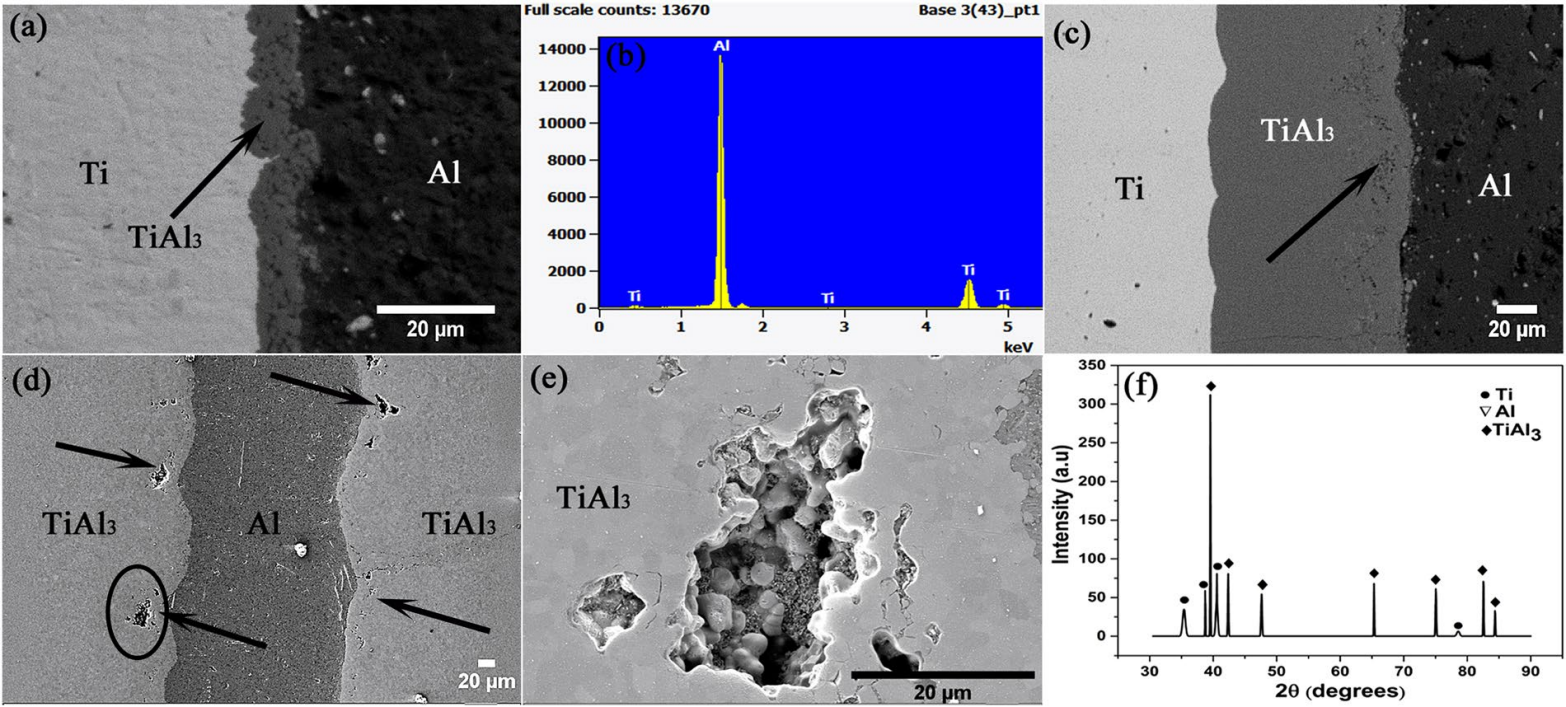
(**a**) BSE mode SEM image of MIL bonded at 575 °C for 6 h (**b**) EDS spectrum of the intermetallic phase (**c**,**d**) SEM image of MILs bonded at 550 °C & 36 h and 575 °C & 36 h respectively showing Kirkendall pores (**e**) SEM image of Kirkendall pore under high magnification (**f**) XRD pattern of MIL annealed at 575 °C for 48 h.

Figure 1a shows the back scattered electron (BSE) mode SEM image of the MIL bonded at 575 °C for 6 h duration. The bright and dark regions in the image represent the Ti and Al sheets used for multi-laminated Ti/Al diffusion couple. The grey coloured layer in between the Ti and Al sheets denotes the product phase that was formed due to the inter-diffusion of Ti and Al atoms. Through EDS analysis, the composition of the product phase was found to be nearly 25 at% Ti and 75 at.% Al, which reveals that the phase formed was TiAl_3_ intermetallic compound and its growth is mainly facilitated due to low free energy of formation^6,13–15^. Figure 1b shows a typical EDS spectrum obtained from the product phase of the MIL bonded at 575 °C for 6 h. The TiAl_3_ phase was found to grow with further annealing at both the annealing temperatures, with increase in annealing time, by which complete consumption of the Al layer was achieved after 48 h annealing at 575 °C. Figure 1c,d shows the SEM images exhibiting the TiAl_3_ layer growth in the MILs annealed for 36 h duration at temperatures 550 and 575 °C respectively. It can be seen from the Fig. 1c that the Ti/TiAl_3_ interface is smoother than Al/TiAl_3_ interface and fine pores are found near the Al/TiAl_3_ interface. These pores are considered to be Kirkendall pores which are formed mainly due to the difference in the diffusivities of the bonding materials. In Ti/Al diffusion couple, the diffusion rates of Ti and Al atoms are different due to the difference in the melting temperature of the diffusing species. The pore formation is also directly associated with vacancy motion as unequal flow of Ti and Al atoms towards either side results in equivalent flow of vacancy to the net flow of atoms^16^. The arrows in Fig. 1c,d represents the straight line Kirkendall pores in the samples annealed for 36 h duration at bonding temperatures of 550 and 575 °C respectively. It can be observed that both fine and coarse pores are present which are always concentrated near the Al/TiAl_3_ interface. Figure 1e shows the highly-magnified SE image of the Kirkendall pore as encircled in Fig. 1d. The XRD patterns of the sample annealed at 575 °C for 48 h duration is shown in Fig. 1f. Only peaks corresponding to Ti and TiAl_3_ were identified during the analysis, as Al was fully consumed during the annealing process. It is evident from the XRD results that the intermetallic phase formed is TiAl_3_. The presence of any phase gradient in the intermetallic layer was analysed using EDS line scan and area mapping. The line scan across the intermetallic layer revealed that there is no concentration gradient of Ti and Al in the intermetallic region (See Supplementary Fig. S2(a)). The elemental area map over the TiAl_3_ layer exhibited the density and elemental distribution of Al is higher than Ti which evidently confirms the TiAl_3_ phase formation (See Supplementary Fig. S2). To, support the claim of dominant diffusion of Al towards Ti side, it is important to calculate the diffusion parameters for Ti and Al in the TiAl_3_ intermetallic region.

### Diffusion Parameters

**Figure 2:**
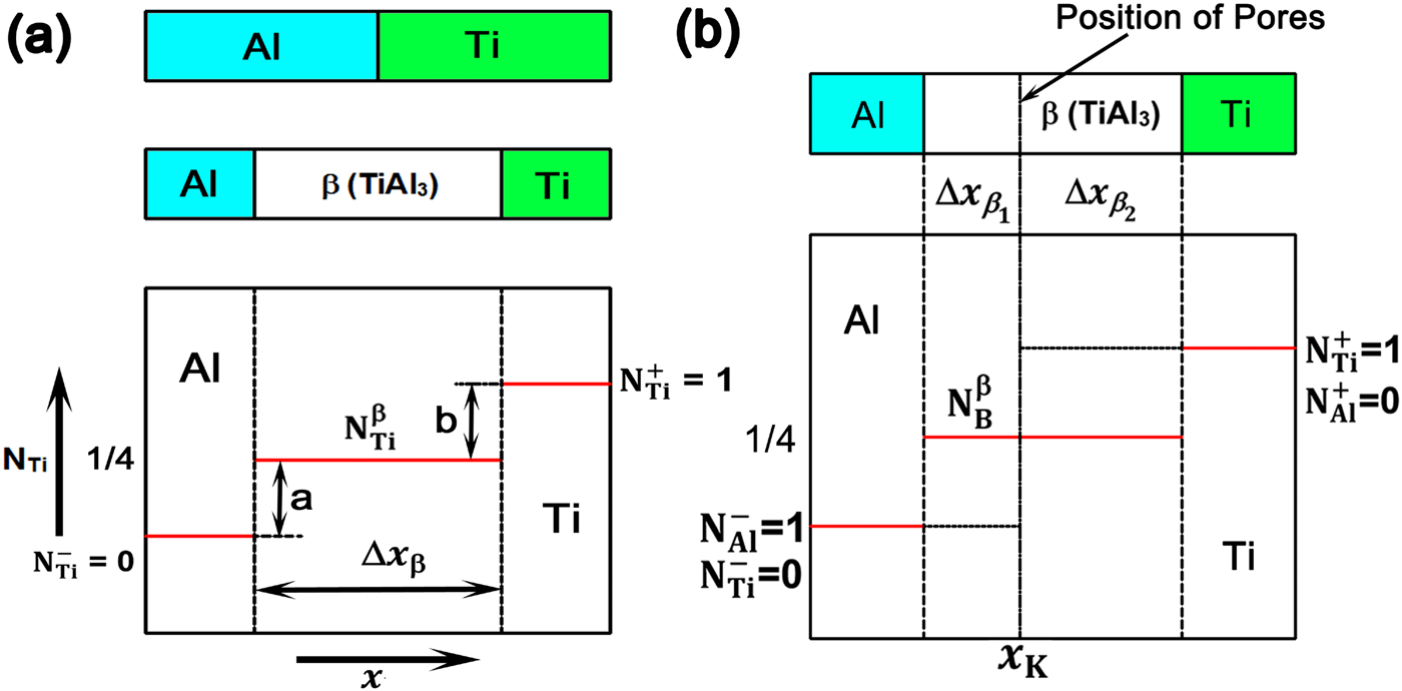
Schematic of composition profile of Ti/Al diffusion couple used for the calculation of (**a**) Integrated diffusion co-efficient of TiAl_3_ and (**b**) Ratio of tracer diffusion co-efficient.

The integrated interdiffusion co-efficient of Al and Ti intermixing can only be calculated over unknown composition gradient of TiAl_3_ in the MILs annealed at 550 and 575 °C for 36 h duration, as TiAl_3_ exists over a narrow homogeneity range without any concentration gradient, using equation (1) derived by Aloke Paul *et al*.^17^ based on Wagner’s approach^12^.

1 $${\tilde{D}}_{int}^{\beta }={\int }_{{N}_{B}^{{\beta }_{1}}}^{N\frac{{\beta }_{2}}{B}}\tilde{D}\,d{N}_{B}$$

where ${\tilde{D}}_{int}^{\beta }$ is the integrated interdiffusion co-efficient of the phase of interest integrated over unknown homogeneity range of the phase, ${N}_{B}={N}_{B}^{{\beta }_{2}}-{N}_{B}^{{\beta }_{1}}$ is the unknown homogeneity range of phase of interest and $\tilde{D}$ is the interdiffusion co-efficient. Since TiAl_3_ is the only intermetallic phase formed as a result of inter-diffusion of Al and Ti atoms which has narrow homogeneity range^13^ and there is no influence of other phases on the growth of TiAl_3_, the contribution of other phases in equation (1) can be neglected and rewritten as

2 $${\tilde{D}}_{int}^{\beta }=\frac{({N}_{B}-{N}_{B}^{-})\,({N}_{B}^{+}-{N}_{B}^{\beta })}{{N}_{B}^{+}-{N}_{B}^{-}}\,\frac{{({{\Delta }}_{{x}_{\beta }})}^{2}}{2t}=\frac{({N}_{Ti}^{TiA{l}_{3}}-{N}_{Ti}^{-})\,({N}_{Ti}^{+}-{N}_{Ti}^{TiA{l}_{3}})}{{N}_{Ti}^{+}-{N}_{Ti}^{-}}\,\frac{{({{\Delta }}_{{x}_{TiA{l}_{3}}})}^{2}}{2t}$$

where ${\tilde{D}}_{int}^{TiA{l}_{3}}$ is the integrated interdiffusion co-efficient of Ti and Al in TiAl_3_ phase, β is the TiAl_3_ phase, ${{\Delta }}_{{x}_{TiA{l}_{3}}}$is the layer thickness of the TiAl_3_ in the Ti/Al diffusion couple, ${N}_{Ti}^{TiA{l}_{3}}$ is atomic fraction of Ti in the TiAl_3_ phase, ${N}_{Ti}^{+}$ and ${N}_{Ti}^{-}$ is the atomic fraction of Ti on the Ti and Al sides respectively in the diffusion couple. The values of the parameters involved in equation (2) can be obtained from the composition profile across Ti/TiAl_3_/Al layer using EDS analysis. From Al-Ti phase diagram^13^, it can be seen that TiAl_3_ exists as a line compound with 25 at.% of Ti. Hence, ${N}_{Ti}^{TiA{l}_{3}}$ is equal to 0.25. ${N}_{Ti}^{-}$ is the atomic fraction of Ti on the Al side which is equal to zero and ${N}_{Ti}^{+}$ is the atomic fraction of Ti on Ti side which is equal to one. The schematic of the diffusion couple before and after bonding and the composition profile are shown in Fig. 2a. Using equation (2), the integrated diffusion co-efficient for intermixing of Al and Ti in MIL bonded at 550 °C and 575 °C for 36 h duration was found to be 5.882 × 10^−15^ m^2^/s and 34.42 × 10^−15^ m^2^/s respectively. The values indicate that the integrated diffusion co-efficient is higher for the MILs annealed at 575 °C.

For better understanding of relative diffusion between Al and Ti atoms, it is necessary to calculate the intrinsic diffusivities of the individual Al and Ti components. It is difficult to calculate the absolute intrinsic diffusivities of individual Ti and Al, as the intrinsic diffusivities can only be measured at a composition indicated by inert markers/tracer. So, the basic criteria to calculate the tracer diffusivities is to find the position of the tracer in the diffusion couple. In this case, as there was no tracer used during the bonding of Al and Ti, the presence of straight line Kirkendall pores near the Al/TiAl_3_ interface as shown in Fig. 1c,d is considered to be the marker plane (Aloke Paul (2014)) for intrinsic diffusivity calculation.

The ratio of the tracer diffusivities was calculated using the equation (3) obtained from Aloke Paul *et al*.^17^

3 $$\begin{array}{lll}\frac{{D}_{B}^{\ast }}{{D}_{A}^{\ast }}=[\frac{{N}_{B}^{+}{\Phi }-{N}_{B}^{-}{\Psi }}{-{N}_{A}^{+}{\Phi }+{N}_{A}^{-}{\Psi }}] & {\rm{or}} & \frac{{D}_{Ti}^{\ast }}{{D}_{Al}^{\ast }}=[\frac{{N}_{Ti}^{+}{\Phi }-{N}_{Ti}^{-}{\Psi }}{-{N}_{Al}^{+}{\Phi }+{N}_{Al}^{-}{\Psi }}]\end{array}$$

4 $${\rm{\Phi }}=\frac{{N}_{B}^{\beta }-{N}_{B}^{-}}{{v}_{m}}{\rm{\Delta }}{x}_{{\beta }_{1}}=\frac{{N}_{Ti}^{TiA{l}_{3}}-{N}_{Ti}^{-}}{{v}_{m}^{TiA{l}_{3}}}{\rm{\Delta }}{x}_{{\beta }_{1}}$$

5 $${\rm{\Psi }}=\frac{{N}_{B}^{-}-{N}_{B}^{\beta }}{{v}_{m}}{\rm{\Delta }}{x}_{{\beta }_{2}}=\frac{{N}_{Ti}^{-}-{N}_{Ti}^{TiA{l}_{3}}}{{v}_{m}^{TiA{l}_{3}}}{\rm{\Delta }}{x}_{{\beta }_{2}}$$

where ${D}_{Al}^{\ast }$ and ${D}_{Ti}^{\ast }$ are tracer diffusion co-efficients for Al and Ti, ${N}_{Ti}^{-}\,and\,{N}_{Ti}^{+}$ are the atomic fractions of Ti on the left and right hand side of the diffusion couple, ${N}_{Al}^{-}\,and\,{N}_{Al}^{+}$ are the atomic fractions of Al on the left and right hand side of the diffusion couple, ${v}_{m}^{TiA{l}_{3}}$ is the molar volume of TiAl_3_ phase, ${\rm{\Delta }}{x}_{{\beta }_{1}}$and ${\rm{\Delta }}{x}_{{\beta }_{2}}$ are the position of the marker from Al and Ti side respectively. Figure 2b shows the schematic of the composition profile used for the estimation of ratio of tracer diffusivities. Using the equations (3–5), the ratio of tracer diffusivities $\frac{{D}_{Al}^{\ast }}{{D}_{Ti}^{\ast }}$ was found to be 17 and 22.6 respectively for the MILs annealed at 550 °C and 575 °C for 36 h duration. This denotes that the diffusivity of Al is higher than Ti in both the MILs.

The absolute values of the tracer diffusion co-efficient are calculated using the equation (6) from Aloke Paul *et al*.^17^

6 $${\tilde{D}}_{int}^{\beta }=-\,({N}_{A}^{\beta }\,{D}_{B}^{\ast }+{N}_{B}^{\beta }\,{D}_{A}^{\ast })\,\frac{{\rm{\Delta }}f{g}_{\beta }}{RT}=-\,({N}_{Al}^{TiA{l}_{3}}\,{D}_{Ti}^{\ast }+{N}_{Ti}^{TiA{l}_{3}}\,{D}_{Al}^{\ast })\,\frac{{\rm{\Delta }}f{g}_{TiA{l}_{3}}}{RT}$$

where ${N}_{Al}^{TiA{l}_{3}}$ and ${N}_{Ti}^{TiA{l}_{3}}$ are the atomic fraction of Al and Ti present in the TiAl_3_ phase respectively, ${\rm{\Delta }}f{g}_{TiA{l}_{3}}$ is the free energy for the formation of TiAl_3_, R is the universal gas constant and T is the bonding temperature. The free energy for the formation of TiAl_3_ was obtained from the work of Peng *et al*.^6^ and is used for the calculation. Table 1 shows the calculated absolute values of tracer diffusivity and integrated diffusion co-efficient for the Ti/Al diffusion couple annealed at 550 °C and 575 °C for 36 h duration respectively. It is now evident from the tracer diffusion co-efficient calculation, that Al is diffusing dominantly which is almost 20 times faster than Ti near the marker plane in Ti/Al diffusion couple.

**Table 1:** Calculated values of integrated diffusion co-efficient, ratio of tracer diffusion co-efficient and absolute values of tracer diffusion co-efficient.

Conditions	${{\boldsymbol{v}}}_{{\boldsymbol{m}}}^{{\boldsymbol{\beta }}}$ (×10^−5^ m^3^/mole)	Integrated Diffusion Co-efficient, ${\tilde{{\boldsymbol{D}}}}_{{\boldsymbol{int}}}^{{\boldsymbol{\beta }}}$ (x 10^−15^ m^2^/s)	Tracer Diffusion Co-efficient Ratio, $\frac{{{\boldsymbol{D}}}_{{\boldsymbol{Al}}}^{\ast }}{{{\boldsymbol{D}}}_{{\boldsymbol{Ti}}}^{\ast }}$	Tracer Diffusion Co-efficient (×10^−15^ m^2^/s)	
				${{\boldsymbol{D}}}_{{\boldsymbol{Al}}}^{\ast }$	${{\boldsymbol{D}}}_{{\boldsymbol{Ti}}}^{\ast }$
550 °C, 36 h	3.845	5.882	17	4.297	0.254
575 °C, 36 h	3.845	34.42	22.6	27.133	1.202

### Diffusion, Nucleation and Growth

**Figure 3:**
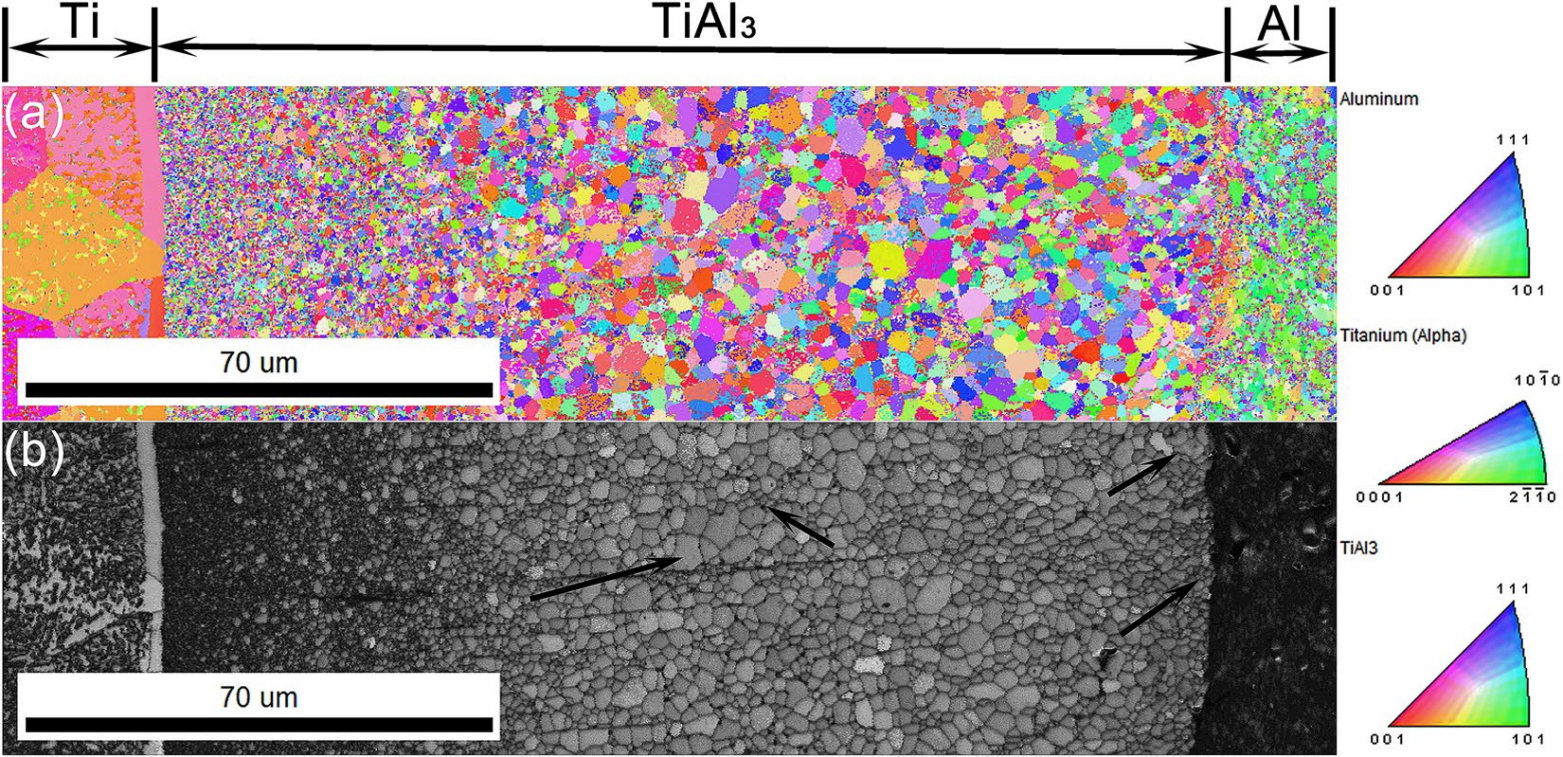
OIM micrographs of MIL annealed at 550 °C for 36 h duration (**a**) IPF Z Map (**b**) Band contrast image.

In Ti/Al diffusion couple, the initial TiAl_3_ intermetallic phase nucleates as a result of reaction between Ti and Al atoms along the Ti/Al interface. In the present work, the grey coloured coarse globular like TiAl_3_ phase (as shown in Fig. 1a) shows the initial stage of nucleation and the couple is further employed for different annealing runs to study the nucleation and growth phenomenon of TiAl_3_ along Ti/TiAl_3_ to Al/TiAl_3_ interfaces. Figure 3a,b shows the IPF Z map and band contrast image of such MIL annealed at 550 °C for 36 h obtained through EBSD. It can be observed from Fig. 3b that the distribution of TiAl_3_ grains is varying along the intermetallic layer thickness starting from Ti/TiAl_3_ to Al/TiAl_3_ interface.

The TiAl_3_ grains near the Ti/TiAl_3_ interface appears to be nearly nano-sized, which spans over a definite length starting from that interface, following, as indicated by the arrows at the centre portion in Fig. 3b, larger grains are concentrated over that region representing grain growth. While at the Al/TiAl_3_ interface, as shown by the arrows near that interface in Fig. 3b, the grain size again gets reduced than at the centre portion but relatively larger than the grains at Ti/TiAl_3_ interface. This depicts that the TiAl_3_ grains nucleated at Ti/TiAl_3_ interface appears to be smaller and uniform than at the other end. These fine grains of TiAl_3_ phase on both the interfaces can be considered as the TiAl_3_ nuclei which are nucleated as a result of diffusion of both Ti and Al across the intermetallic layer and through reactions at the respective interfaces.

**Figure 4:**
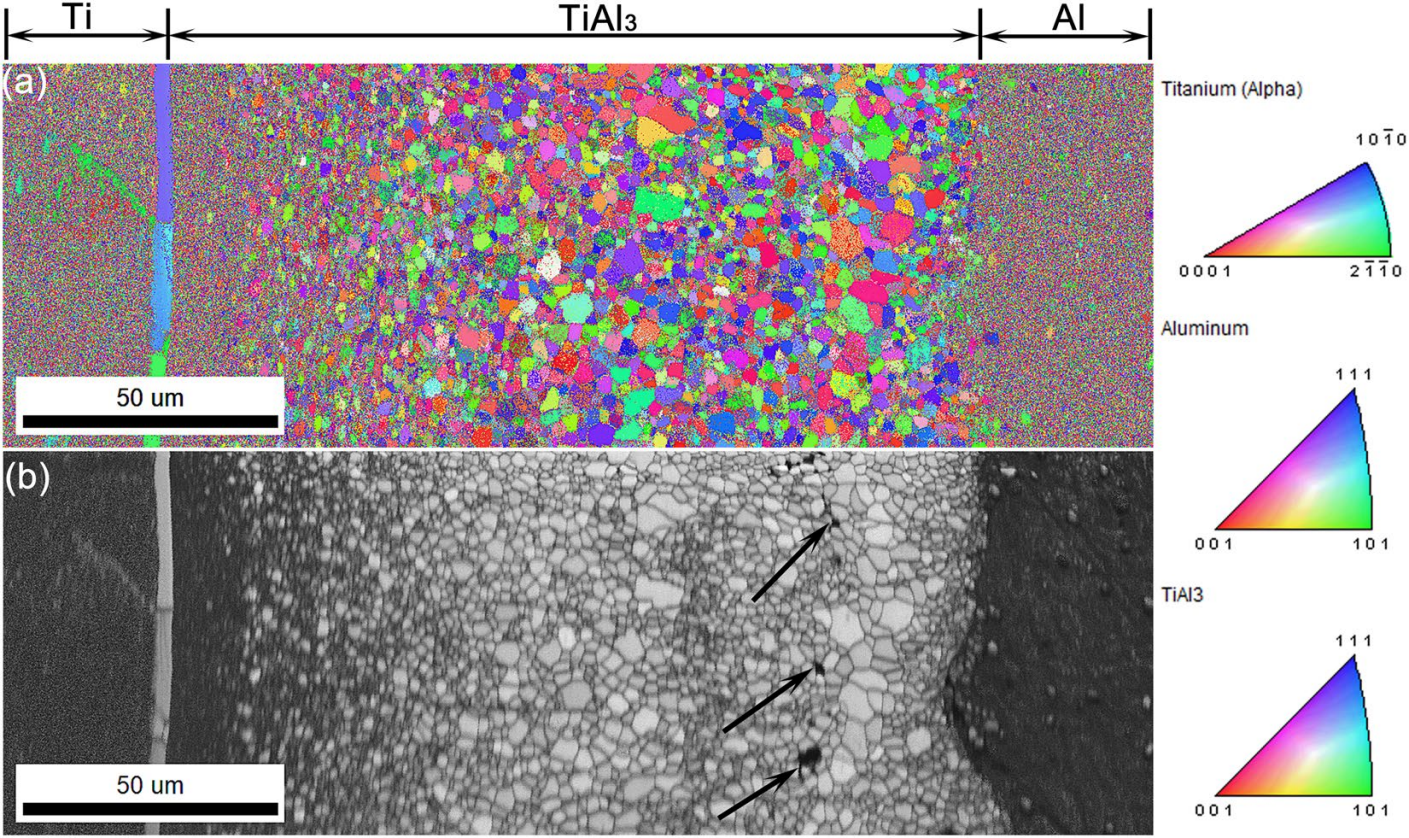
OIM micrographs of MIL annealed at 575 °C for 36 h duration (**a**) IPF Z Map (**b**) Band contrast image.

Similar pattern of TiAl_3_ grain structure was also observed in the MIL annealed at 575 °C for 36 h which is shown in Fig. 4a,b. Here also, as it can be seen from Fig. 4b, the TiAl_3_ nuclei at Ti/TiAl_3_ interface appears to be nearly nano-sized than the grains near Al/TiAl_3_ interface depicting dominant nucleation and growth. Also, the centre region is almost concentrated of larger grains than at the interfaces. The straight line of Kirkendall pores are visible in the grey scale image which are denoted by the arrows in Fig. 4b. These Kirkendall pores are always observed to be present near the Al/TiAl_3_ interface and their direction of movement of these pores is towards the Al side (See Supplementary image S3) which is mainly due to high diffusion flux of Al than Ti. It is evident from the calculated intrinsic diffusion co-efficient of Al and Ti. Finally, if complete consumption of Al is achieved during annealing runs, these pores are expected to concentrate over the centre portion of TiAl_3_ intermetallic layer. We can even observe the presence of larger grains near the Kirkendall pores indicating grain growth and minimal TiAl_3_ nucleation at the Al/TiAl_3_ interface. It can be ascertained that the EBSD analysis of the MILs annealed at both the conditions as shown in Figs 3 and 4 reveals similar TiAl_3_ grain structure and growth phenomenon throughout the entire intermetallic layer thickness.

**Figure 5:**
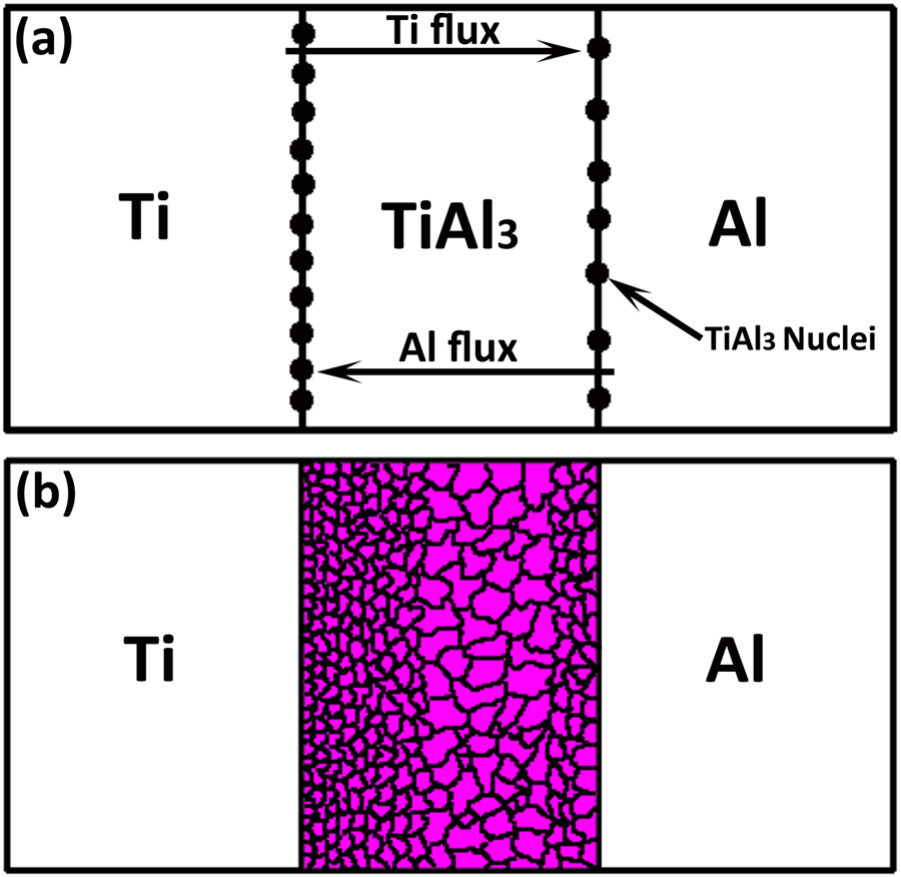
Schematic of (**a**) Ti/Al diffusion process and (**b**) TiAl_3_ grain structure.

## Discussion

On discussing the TiAl_3_ growth phenomenon in Ti/Al couple, diffusion kinetics plays an important role in determining the interface where the faster growth of TiAl_3_ takes place, as the individual Ti and Al atoms diffuse across the intermetallic layer to react with the respective interfaces. It is expected that Ti(Al) and Al(Ti) solid solution formation precedes the TiAl_3_ nucleation at both Ti/TiAl_3_ and Al/TiAl_3_ interfaces. From Ti-Al binary phase diagram^13^, it can be ascertained that the Ti(Al) solid solution exists over a wide composition range whereas Al(Ti) solid solution regime appears to be narrow signifying higher solubility of Al in Ti rather than Ti in Al. With the above phenomenon, Xu *et al*.^10^ claimed earlier saturation of Al(Ti) solid solution than Ti(Al), due to less solubility resulting in faster nucleation of TiAl_3_ nuclei along Al/TiAl_3_ interface and supposed that the extended solubility of Al in Ti is for Ti_3_Al nucleation rather than TiAl_3_. It was also expected that the distribution of TiAl_3_ nuclei near Al/TiAl_3_ interface must be fine and uniform, while on the other side it would be relatively coarse^10^.

In the present study, the distribution of these TiAl_3_ nuclei/grains are exactly opposite, that fine, uniform and densely populated TiAl_3_ nuclei are visible only at the Ti/TiAl_3_ interface whereas relatively larger nuclei are observed at the Al/TiAl_3_ interface as shown in Figs 3 and 4. Also, the formation of Ti(Al) and Al(Ti) solid solution phases are not visible along the interfaces. Figure 5a,b shows the schematic of the Ti/Al diffusion process and TiAl_3_ grains distribution in which black dots represents the TiAl_3_ nuclei. Considering the diffusion kinetics in the present system, as Al diffuses faster (at the employed temperatures) than Ti, more number of Al atoms are expected to present near the Kirkendall marker plane than Ti. These Al atoms, with respect to the basic diffusion law tends to move from high to low concentration region i.e. it always move from Al to Ti side through the intermetallic layer, ensuring high flow of Al atoms towards Ti/TiAl_3_ interface for reaction. In other words, the chemical reactivity/potential for Al atoms to interact with Ti/TiAl_3_ is higher than at the Al/TiAl_3_ interface. As the activation energy for grain boundary diffusion is generally lower than lattice diffusion, the TiAl_3_ grain boundaries acts as faster diffusion channels for the moving Al atoms.

In contrast, even if grain boundary diffusion dominates, relatively low diffusivity of Ti towards Al is expected (annealing temperature is almost 1/3^rd^ of melting temperature of Ti), resulting in less availability of Ti atoms at Al/TiAl_3_ interface for TiAl_3_ nucleation. This strongly suggests, although Al(Ti) solid solution saturates earlier than Ti(Al), the limited availability of Ti atoms for interaction with Al at the Al/TiAl_3_ interface restricts faster nucleation, ensuing relatively coarse and not so densely populated TiAl_3_ nuclei. On the other hand, at the Ti side, as solubility of Al in Ti is higher, TiAl_3_ nuclei should form at the Ti grain boundaries which facilitates faster diffusion of Al and earlier saturation of Ti(Al) solid solution when the solubility gets locally exceeded, than at the lattice. The activation energy for such grain boundary diffusion controlled (~33 kJ mol^−1^) TiAl_3_ intermetallic layer growth is always lower than lattice diffusion controlled growth (~295 kJ mol^−1^)^9^. This concludes that abundant availability of Al atoms at the Ti/TiAl_3_ interface facilitates faster TiAl_3_ nucleation than at Al/TiAl_3_ side resulting in tri-modal grain structure of TiAl_3_ phase. Figure 5b shows the schematic of TiAl_3_ grain structure after annealing.

**Figure 6:**
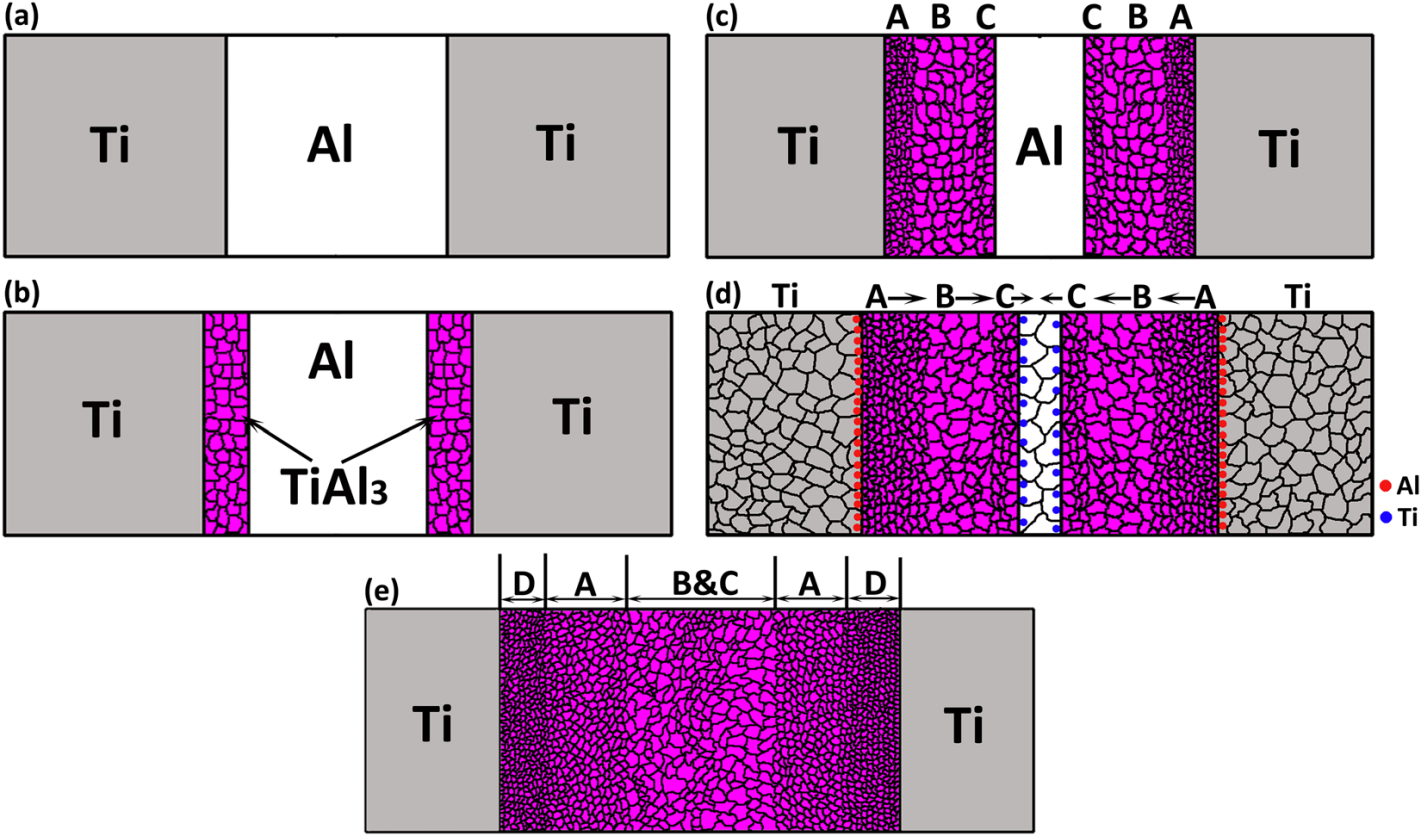
Schematic of TiAl_3_ growth phenomenon in a Ti/Al/Ti tri-layer system (**a**) Ti/Al/Ti stacking (**b**) Initial TiAl_3_ growth (**c**) Tri-modal TiAl_3_ grain structure (**d**) TiAl_3_ growth process in Ti/Al/Ti Tri-layer system (**e**) Final TiAl_3_ grain structure in the Ti/Al/Ti system after Al consumption.

**Figure 7:**
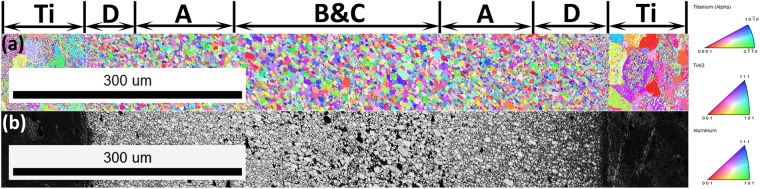
OIM micrographs of MIL annealed at 575 °C for 48 h duration (**a**) IPF Z Map (**b**) Band contrast image.

A better understanding of nucleation and growth phenomenon of TiAl_3_ intermetallic phase can be achieved by considering a Ti/Al/Ti tri-layer system rather than Ti/Al diffusion couple. Figure 6 shows the schematic of TiAl_3_ growth phenomenon in a Ti/Al/Ti tri-layer system. Figure 6a shows the stacking of Ti/Al/Ti sheets of equal thickness before bonding. While diffusion bonding, the individual Ti and Al atoms inter-diffuse over time to form initial TiAl_3_ intermetallic phase along the Ti/Al interface which is shown in Fig. 6b. After annealing, a tri-modal TiAl_3_ grain structure was observed between Ti and Al sheets, consisting of nearly nano-sized TiAl_3_ grains/nuclei near Ti/TiAl_3_ interface, coarse grain structure at the centre part and relatively small TiAl_3_ grains/nuclei at the Al/TiAl_3_ interface. Figure 6c shows the schematic of Ti/Al/Ti tri-layer system with tri-modal TiAl_3_ grain structure, which are represented as region A, B and C respectively. As Al diffuses faster than Ti, we can expect huge mass transport from Al to Ti side i.e. more number of Al atoms will be moved towards Ti side. Whereas relatively lower diffusivity of Ti does not ensure equivalent mass transport towards the Al side ensuring net mass transport of atoms towards Ti. This is illustrated in Fig. 6d by the presence of red (Al atoms) and blue (Ti atoms) dots of different densities at the Ti/TiAl_3_ and Al/TiAl_3_ interfaces. As relatively faster growth of TiAl_3_ nuclei occurs at Ti/TiAl_3_ interface than at Al/TiAl_3_ side, the growth of new TiAl_3_ nuclei at Ti/TiAl_3_ side will shift the already present TiAl_3_ grains i.e. Region A towards Al side, simultaneously shifting the regions B and C towards Al side. The arrows near the denoted regions A, B and C in Fig. 6d represents the shifting direction. When the annealing is continued for further growth of new TiAl_3_ nuclei at Ti/TiAl_3_ side, the regions A, B and C always tend to move towards Al side, such that if complete consumption of Al is achieved in the growth process, intermixing of the regions B and C is expected at the center portion of the intermetallic layer. Figure 6e shows the schematic of TiAl_3_ grain structure after complete consumption of Al sheet. Thus, the final TiAl_3_ grain structure consists of newly grown fine TiAl_3_ nuclei at Ti/TiAl_3_ interface (Region D), Region A with minimal growth shifted towards Al side, adjacent to region D and intermixed regions B and C at the centre portion. The above-mentioned grain structure was observed in the MIL annealed at 575 °C for 48 h in which Al sheet was completely consumed in the TiAl_3_ growth process. Figure 7a,b shows the IPF Z map and band contrast image of MIL annealed at 575 °C for 48 h obtained through EBSD where the different grain structures are denoted as A, B, C and D. We can observe that the TiAl_3_ grains near both the Ti/TiAl_3_ interfaces are very fine-sized (Region D), with moderately grown TiAl_3_ grains (Region A), adjacent to Region D and the presence of both coarse and fine grains at the centre part (intermixed regions B and C).

This clearly denotes that diffusion kinetics plays an important role in nucleation of TiAl_3_ phase in Ti/Al system which is mainly concentrated along the Ti/TiAl_3_ interface during annealing due to the dominant diffusion of Al towards Ti side. Whereas, TiAl_3_ grains near the Al/TiAl_3_ interface are found to grow during annealing with minimal nucleation due to less diffusion of Ti towards Al side. And if, solid solution formation precedes the TiAl_3_ nucleation, the formation of Ti(Al) solid solution near Ti/TiAl_3_ interface is in favour of growth of TiAl_3_ than Ti_3_Al due to low free energy of formation of TiAl_3_^6^. Thus, in contrast to the viewpoints of Xu *et al*.^10^, the low solubility of Ti in Al did not favour faster nucleation of TiAl_3_ near Al/TiAl_3_ interface and it is faster only at the Ti/TiAl_3_ interface.

In conclusion, a novel growth phenomenon for TiAl_3_ intermetallic phase in Ti/Al diffusion couple was proposed based on diffusion kinetics. The dominant diffusion of Al towards Ti in Ti/Al binary diffusion couple was strongly revealed through the interdiffusion and intrinsic diffusion co-efficient calculations based on Wagner’s approach. The nucleation site of TiAl_3_ intermetallic phase was found to be concentrated mostly along Ti/TiAl_3_ interface rather than Al/TiAl_3_ due to dominant diffusion of Al towards Ti side which was supported by the diffusion parameter calculations. Thus, the present work provides better phenomenon and clears the ambiguity involved in the nucleation and growth sites of TiAl_3_ intermetallic phase in Ti/Al diffusion system.

## Electronic supplementary material

Figs S1, S2 and S3
