# Supplementary material for: Nucleation and growth of TiAl3 intermetallic phase in diffusion bonded Ti/Al Metal Intermetallic Laminate
Source: Sci Rep. 2018 Nov 14;8:16797. doi: 10.1038/s41598-018-35247-0 (PMC6235842; doi:10.1038/s41598-018-35247-0)
Supplement: Supplementary file 1 — Figs S1, S2 and S3 [file 41598_2018_35247_MOESM1_ESM.pdf]

Supplemetary Information

Nucleation and growth of $TiAl_3$ intermetallic phase in diffusion bonded Ti/Al Metal Intermetallic Laminate

N. Thiyaneshwaran, K. Sivaprasad*, B. Ravisankar

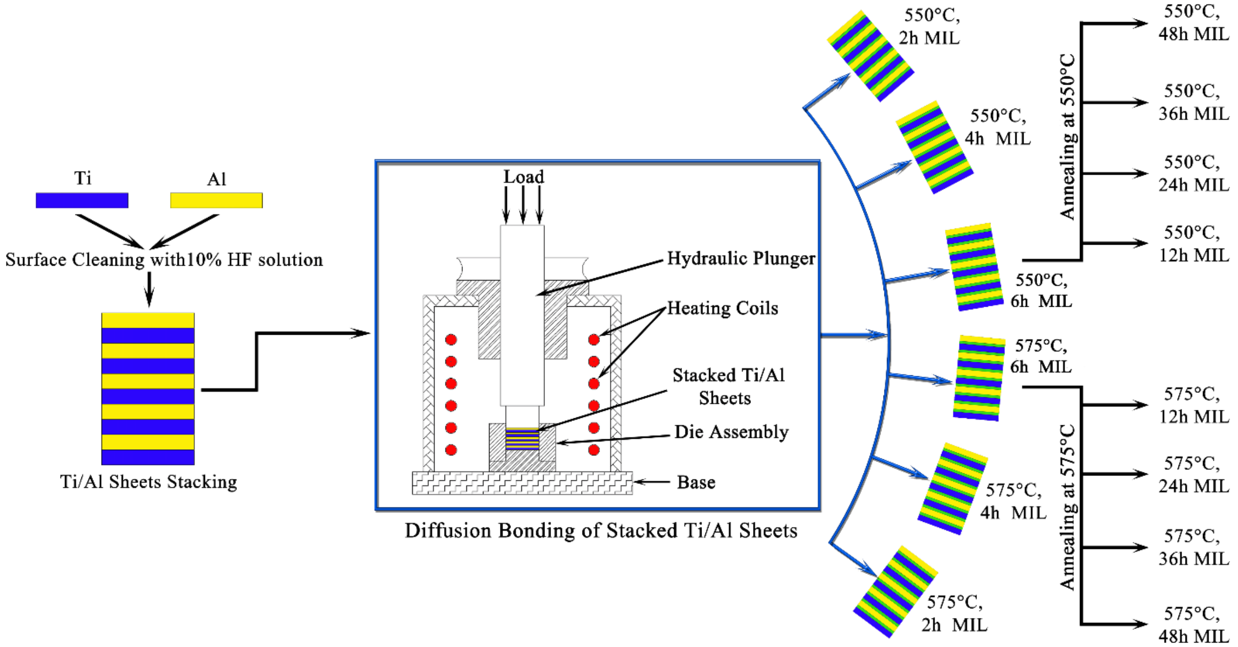

Fig. S1 Schematic diagram showing the Ti/Al metal intermetallic laminates preparation process.

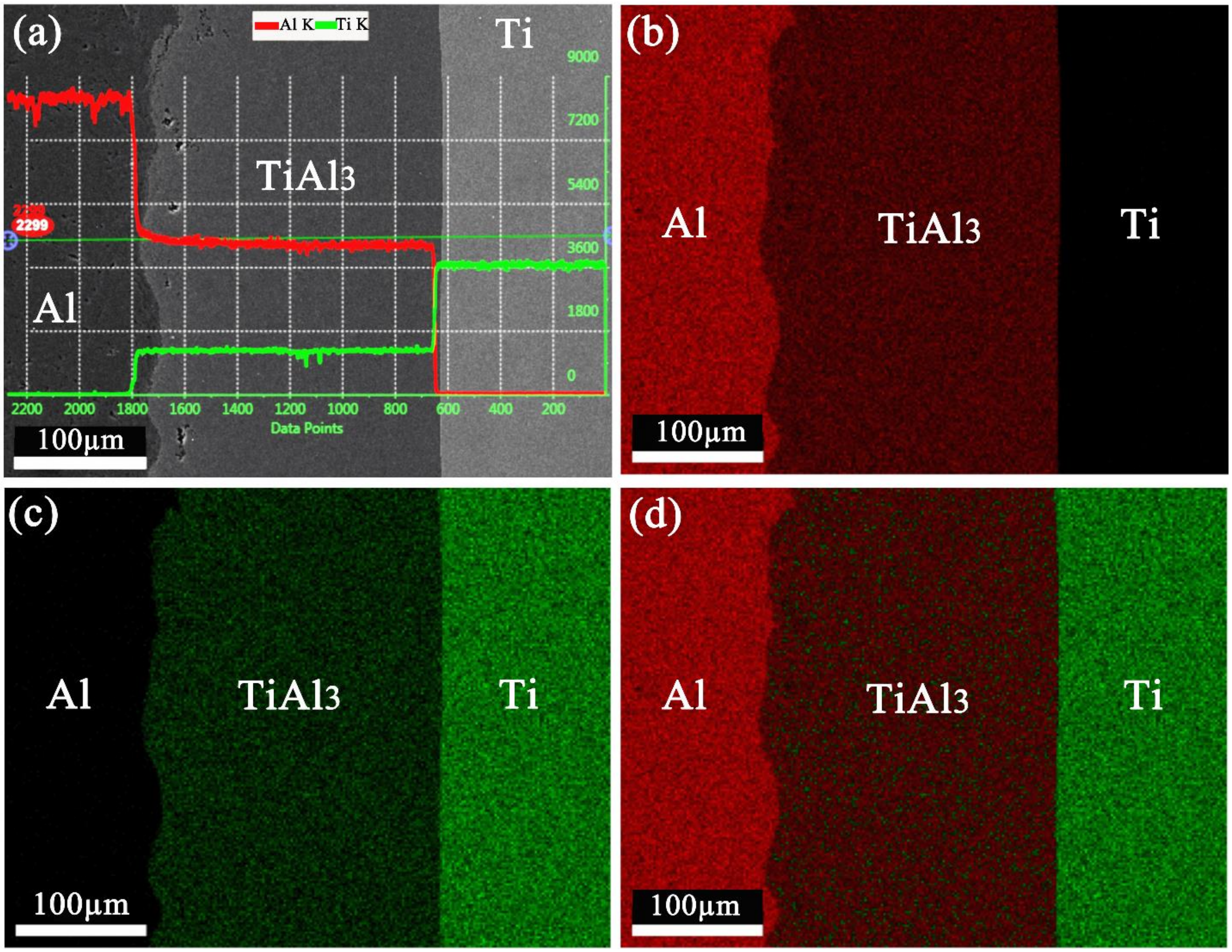

Fig. S2 SEM images showing Line Scan and elemental mapping in MIL annealed at 575C for 36h duration (a) Concentration profile, (b) Al distribution, (c) Ti distribution and (d) Ti and Al distribution in the sample.

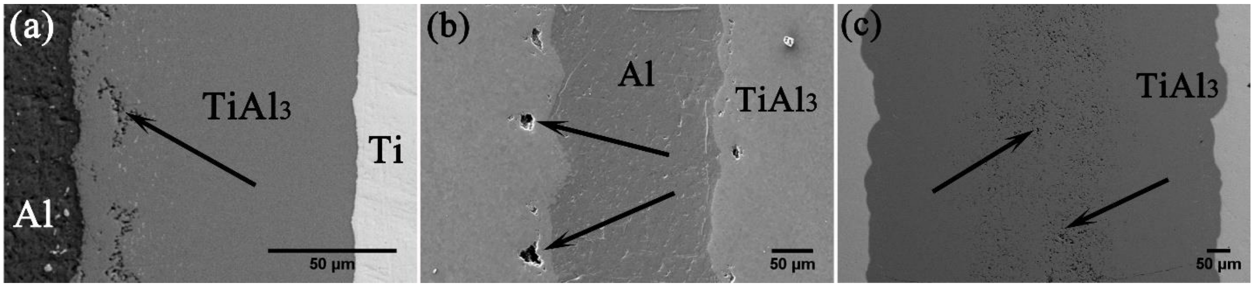

Fig. S3 SEM images showing the consecutive position of kirkendall pores in the MILs annealed at (a) 575°C & 24h, (b) 575°C & 36h and (c) 575°C & 48h duration respectively. The arrows in the images shows the kirkendall pores in the intermetallic layer.
